# Selection of reference genes for qPCR normalization in buffalobur (*Solanum rostratum* Dunal)

**DOI:** 10.1038/s41598-019-43438-6

**Published:** 2019-05-06

**Authors:** Dandan Zhao, Xu Wang, Jingchao Chen, Zhaofeng Huang, Heqiang Huo, Cuilan Jiang, Hongjuan Huang, Chaoxian Zhang, Shouhui Wei

**Affiliations:** 1 Institute of Plant Protection, Chinese Academy of Agricultural Sciences, Beijing, China; 2 Mid-Florida Research and Education Center, University of Florida, Institute of Food and Agricultural Sciences, Apopka, Florida United States of America

**Keywords:** Plant stress responses, Molecular biology

## Abstract

Buffalobur (*Solanum rostratum* Dunal), which belongs to the Solanaceae family, is a worldwide noxious invasive weed and is listed as one of the top 10 alien invasive species in China. It is harmful to humans and livestock because the entire plant is covered with spines containing toxins. Many studies have analysed the gene expression in this weed species under different stress conditions using quantitative real-time PCR (qPCR). However, until now, there has been no report on suitable reference genes in buffalobur. Herein, 14 candidate reference genes were selected and evaluated for their expression stability in buffalobur in different tissues, at different developmental stages, and in response to several stress conditions using the geNorm, NormFinder, BestKeeper and RefFinder statistical algorithms. The results showed that *EF1α*, *ACT* and *SAND* are suitable reference genes across all samples tested. We recommend the normalization of target gene expression under different experimental conditions using these three genes together. Validation of selected reference genes was achieved by assessing the relative expression levels of *P5CS* and *GI*. This work identified the appropriate reference genes for transcript normalization in buffalobur, which will be helpful in future genetic studies of this invasive weed.

## Introduction

Buffalobur (*Solanum rostratum* Dunal) is native to North America and is ranked as a highly invasive weed species across the world^1^. Buffalobur poses a serious threat to local biodiversity and agro-ecosystems. The aggressive growth of this weed results in devastating damage to crop production^2^. Additionally, buffalobur is a crucial intermediate host for a wide range of pests and diseases, which threaten the health of crops^3^. Moreover, this weed species is harmful to humans and livestock because its leaves, stems and calyx are covered with spines containing toxins^3^. Eradication of this weed using conventional control measures including manual, mechanical, or chemical methods is difficult. Therefore, new and innovative approaches are being explored to control this weed. Measuring gene expression will help form weed-control approaches, and quantitative gene expression measurement requires appropriate reference genes.

Due to its advantages of high sensitivity and specificity, qPCR has been widely used to quantify gene expression to discover the genetic basis of physiological patterns during the plant life cycle^4^. Attaining precision in qPCR-based analysis depends on the selection of a suitable reference gene in experimental sets^5^. The expression level of the appropriate internal control gene should remain relatively constant and should not change significantly across experimental conditions, types of tissues, developmental stages or stress treatments^6,7^; however, in practice, no gene exhibits fully stable expression throughout all growth stages and experimental conditions. It has been suggested that multiple reference genes can achieve accurate normalization^8^. There is general agreement that the expression stability of candidate genes should be validated prior to initiating normalization studies using qPCR in a particular species.

There have been studies on the selection and validation of reference genes in many Solanaceae plants, such as pepper (*Capsicum annuum* L.)^9^, potato (*Solanum tuberosum* L.)^10^, eggplant (*Solanum melongena* L.)^11^, tomato (*Lycopersicon esculentum* Mill.)^12^, *Lycium barbarum* L. and *L*. *ruthenicum* Murray^13^. However, until now, no appropriate reference gene has been identified for qPCR analysis in buffalobur. In this study, 14 genes, namely, *GAPDH*, *ACT*, *GR*, *UBQ*, *TIP41*, *RPL8*, *eIF*, *DNAJ*, *TUB*, *CYP*, *EF1α*, *PP2Acs*, *RUBP*, and *SAND*, were selected as candidate reference genes for buffalobur. The expression stabilities of these genes were tested with respect to different developmental periods, tissues, abiotic stresses, and hormone stimuli and with glyphosate treatment using geNorm^8^, NormFinder^14^, BestKeeper^15^ and RefFinder^16^ to identify the most stable gene for qPCR normalization in buffalobur.

## Results

### Expression profiles of candidate reference genes

**Figure 1:**
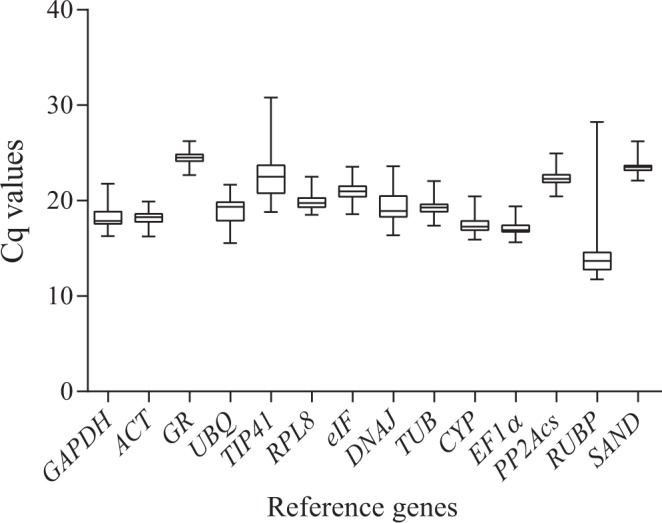
Cq values of 14 reference genes across all samples. The box plots represent the interquartile range, the median, and the maximum and minimum values of each reference gene in all buffalobur samples.

The quantification cycle values (Cq) of the 14 candidate genes across all samples ranged from 11.8 to 30.8, showing a wide range of variation (Fig. 1). The majority of values were between 18 and 23. The Cq values of *TIP41* and *RUBP* ranged from 18.8 to 30.8 and from 11.8 to 28.3, respectively, showing great expression differences. Moreover, the Cq values of *RUBP* in roots were relatively high. According to the Cq values, we can make a preliminary judgement that *TIP41* and *RUBP* might not be suitable candidate reference genes.

### geNorm analysis

The geNorm software identifies the optimal reference gene pair; smaller M values correlate with more stable gene expression. Some studies have established the threshold of M values ≤ 1.0 or 1.5 to identify appropriate reference genes^17^. M values below 0.5 are indicative of strong reference genes. Except for the group of total samples (M = 0.527), the M values of the best pairs in other groups were less than 0.5 in our study (Tables 2, 3 and 4; see Supplementary Fig. S1). For leaves of different developmental stages and different tissues in the fruiting period, the best two gene pairs were *eIF* and *SAND*. *RPL8* and *EF1α* were the most stable gene pair in both the abiotic stress and glyphosate group. For the hormone stimuli group, the best gene pairs were *ACT* and *RPL8*. For the total samples group, the best gene pairs were *ACT* and *PP2Acs*. *TIP41*, *RUBP* and *DNAJ* were low-ranking candidates according to geNorm.

Additionally, geNorm provides the pairwise variation (V_n_/V_n+1_), which determines the minimal number of reference genes to obtain an accurate and reliable normalization. A value of 0.2 is usually considered acceptable. For the total samples group, the V3/4 value of 0.186 indicated that the top three reference genes (*ACT*, *PP2Acs* and *SAND*) would be appropriate to use for normalization; for the other groups, the top two genes were sufficient for normalization because their V2/3 values were all less than 0.15 (see Supplementary Fig. S2).

### NormFinder analysis

The lower the stability value obtained via NormFinder, the more stable the candidate reference gene is. A grouped analysis can be used to evaluate these candidate reference genes with this statistical algorithm. The output includes the best reference gene and the best combination of two reference genes (Tables 2, 3 and 4; see Supplementary Table S3). Notably, the ranking alone cannot identify whether a candidate gene is good, the stability values need to be considered for the assessment. Only when the stability value is low enough, the gene can be considered good. In our study, for leaves of different developmental stages (grouped by stage), the best gene was *ACT* (0.111), and the best combination of genes was *GR* and *eIF* with a stability value of 0.082. For different tissues in the fruiting period (grouped by tissue), *SAND* (0.121) was the best reference gene, and the best pair was *GR* and *PP2Acs* (0.084). *ACT* (0.114) was ranked the top gene for glyphosate-treated samples (grouped by time), and *eIF* and *CYP* were the best combination with a stability value of 0.079. For abiotic stress-treated samples (grouped by stress type), the best gene was *EF1α* (0.051), and the best combination of two genes was *TUB* and *EF1α* (0.036). For samples of hormone stimuli (grouped by ABA and GA), *EF1α* (0.019) was also the best reference gene, and *EF1α* and *SAND* (0.015) formed the best gene pair. For the total samples (grouped by subgroup), the top three genes were *EF1α* (0.065), *ACT* (0.065) and *TUB* (0.072); the best combination of two genes was *GAPDH* and *SAND* with a stability value of 0.046. The ranking of candidate reference genes and best combination of two genes from NormFinder were not identical to those from the geNorm analysis (Tables 2, 3 and 4).

### BestKeeper analysis

Pfaffl proposed that genes with high SD or CV values can be considered inconsistent^15^. In general, a gene with an SD value greater than 1 is not acceptable(Tables 2, 3 and 4). For leaves of different developmental stages, *GR* was the optimal reference gene with an SD value of 0.30. For different tissues in the fruiting period and the group with glyphosate treatment, *PP2Acs* (SD values of 0.40 and 0.26, respectively) was the most stable gene. For the abiotic stress group, *EF1α* (an SD value of 0.42) was the most stable of the candidate reference genes. For the hormone treatment and total samples group, BestKeeper ranked *SAND* as the best reference gene with SD values of 0.18 and 0.49, respectively.

### RefFinder analysis

The stability rankings generated by geNorm, NormFinder and BestKeeper, which are based on different algorithms, differed; but in most cases the 3 or 4 same genes were ranked highly, just in different orders. We used RefFinder to obtain a final ranking based on the outputs of the previous three methods (plus Delta-Ct) (Tables 2, 3 and 4). For leaves of different developmental stages and the glyphosate treatment group, the best gene was *ACT* followed by *EF1α*. For different tissues in the fruiting period, the suitable reference genes were *SAND* and *PP2Acs*. For the abiotic stress groups, the top two genes were *EF1α* and *RPL8*. *SAND* and *EF1α* were the most stable genes in the leaves of hormone treatment groups. For the total samples group, the most stable three reference genes were *EF1α*, *ACT* and *SAND*. *RUBP*, *DNAJ* and *TIP41* had unstable expression in all of the groups.

### Reference gene validation

Delta 1-pyrroline-5-carboxylate synthetase (*P5CS*) is the rate-limiting enzyme in the proline synthesis pathway under stress conditions^18^. GIGANTEA (*GI*) participates in multiple molecular regulatory responses including flowering, circadian rhythm and stress tolerance^19^. The elevated expression of *P5CS* and *GI* improves resistance against salinity and drought stresses. Therefore, *P5CS* and *GI* expression should increase or hold stable rather than decrease. Previous studies found that the *Arabidopsis thaliana* gi mutant is resistant to herbicide^20^, indicating that lower expression of *GI* indicates stronger herbicide tolerance.

**Figure 2:**
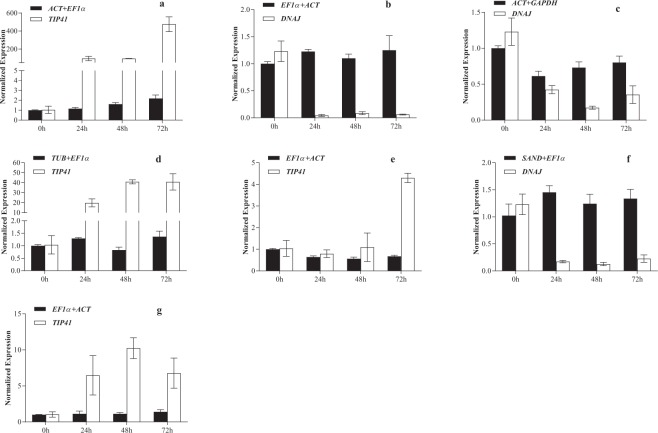
The relative expression patterns of *P5CS* under different treatments and with normalization to different reference genes. (**a**) Glyphosate treatment, (**b**) drought treatment, (**c**) salinity treatment, (**d**) heat treatment, (**e**) cold treatment, (**f**) ABA treatment, and (**g**) GA treatment. The error bars represent the standard error.

**Figure 3:**
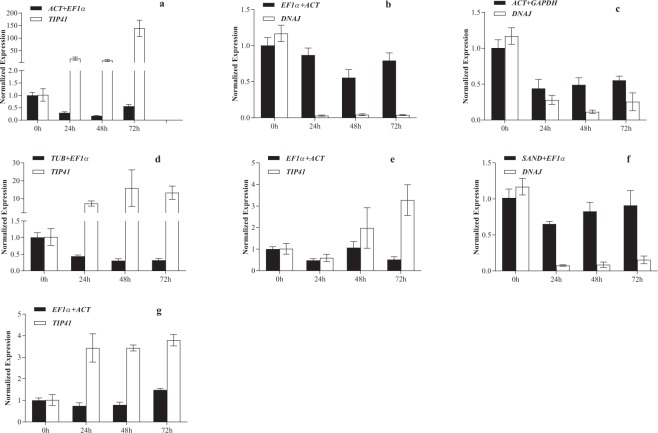
Relative expression of *GI* under different treatments and with normalization to different reference genes. (**a**) Glyphosate treatment, (**b**) drought treatment, (**c**) salinity treatment, (**d**) heat treatment, (**e**) cold treatment, (**f**) ABA treatment, and (**g**) GA treatment. The error bars represent the standard error.

As shown in Figs 2 and 3, the expression patterns of *P5CS* and *GI* were significantly different when using the best reference gene combination for normalization under different stress conditions than when normalized with the least stable reference gene. With the best gene combination, the *P5CS* level at 72 h was 2.2-fold higher than in the untreated sample; with the least stable reference gene, the expression at 72 h was 475-fold higher than in the untreated sample (Fig. 2). Using the stable gene combination, the expression level of *GI* decreased significantly within 48 h of glyphosate treatment, which is consistent with the expected pattern; while using the worst reference gene, its expression dramatically increased (Fig. 3).

In the drought, salinity or abscisic acid (ABA) treatment groups, the expression of *P5CS* and *GI* increased or decreased slightly when using the best reference gene combinations for normalization, and the values ranged from 0.4 to 1.5; when using the least stable reference genes, these expressions decreased by 4 to 30-fold (Figs 2 and 3). The expression patterns of *P5CS* and *GI* normalized by stable reference gene combinations were more in line with the expectations than when they were normalized with the least stable reference genes. For the heat, cold and gibberellin (GA) stress groups, the expression of *P5CS* and *GI* ranged from 0.3 to 1.5 when normalized with the best reference gene combination; the expression normalized by the worst reference genes increased 3 to 40-fold (Figs 2 and 3).

For the relative expression of *P5CS* or *GI*, the coefficients of variation (CV (%)) using the poor reference genes were much higher than those produced by using the best gene combinations (see Supplementary Table S4). This indicates that the normalization with stable reference genes lowers the variability of the raw data, so the selected stable reference genes are acceptable. In our study, the expression normalized with the least stable reference genes *DNAJ* and *TIP41* deviated dramatically from those normalized with the best gene combination. *DNAJ*, a molecular chaperone, responds to stress and maintains protein homeostasis in plant cells^21^. *DNAJ* rises sharply in response to stress, as observed in Figs 2 and 3. Similarly, *TIP41* responds to abiotic stresses via the TOR signalling pathway^22^. Under stress conditions, the acute decrease in *TIP41* levels results in unreasonably higher normalized expression values (Figs 2 and 3).

## Discussion

Buffalobur (*Solanum rostratum* Dunal) is a worldwide noxious invasive weed, and it ranked as one of the top 10 alien invasive species in China^1^. Many studies have focused on understanding the physiological characteristics of this weed, including seed germination and seedling emergence^23^. For genetic research, it is necessary to select stable reference genes to ensure the accuracy of research results. In this study, three software packages and one web tool were used to test the statistical reliability of candidate reference genes.

Notably, the different algorithms evaluating the expression stability of reference genes generated different stable genes due to their different mathematical calculations. One disadvantage of geNorm is that it is likely to select coregulated genes with similar expression profiles, such as genes in the same functional class. When groups are specified, NormFinder considers the inter- and intragroup variations for normalization factor calculation and eliminates artificial selection of coregulated gene values; however, it cannot exclude systematic errors generated from sample preparation^14^. For BestKeeper, input data derived from more than three candidate genes are required for accurate assessment of the stability of each gene^15^. Based on different calculation principles, the stability rankings of three statistical packages were different. RefFinder considers four statistical approaches (geNorm, NormFinder, BestKeeper and Delta-Ct); therefore, we used RefFinder to obtain a comprehensive ranking of gene expression stability.

In our study, the combination of *EF1α*, *ACT* (β-actin) and *SAND* was sufficient to normalize expression levels of target genes in the total samples group. Therefore, it is recommended that *EF1α*, *ACT* and *SAND* should be used together for normalization in various buffalobur experimental samples. *ACT*, which is a cytoskeleton component and cell division regulator^24^, is the most stable gene in leaves of different developmental stages and glyphosate-treated leaves (Tables 2 and 3). *ACT* is also stably expressed in *Hordeum vulgare* L.^25^. In *Descurainia sophia*, actin 7 (*ACT7*) is suitable in most samples under different conditions, and actin 8 (*ACT8*) is the least stable reference gene^26^. *SAND* is the most stable reference gene for different tissues in the fruiting period and in hormone-stimulated leaves (Tables 2 and 3). Similarly, *SAND* is stable in different tomato tissues^12^. In *Stellera chamaejasme* and *Robinia pseudoacacia* L., *SAND* levels are stable under ABA and drought treatments, respectively^27,28^. In *Peucedanum praeruptorum* Dunn, *SAND* and actin 2 (*ACT2*) are the top two most stable reference genes under abiotic stress and hormone treatments and in different tissues^29^. *EF1α*, which participates in the elongation cycle of protein biosynthesis, is the most stable gene for leaves under abiotic stress or hormone stimulation (Tables 2 and 3). In potato, the expression of *EF1α* has been used to normalize expression levels across cold, salt and late blight stresses^10^; in drought and simulated drought environments, *EF1α* performs best out of candidate reference genes^30^. In tomato, *EF1α* is the top-ranked reference gene during N and cold stress^31^. However, for hormone stimuli, *EF1α* ranks behind other candidates in *Diospyros kaki* Thunb and *Hibiscus cannabinus* L.^32,33^. These results suggest that evaluation of reference gene expression stability is indispensable prior to the analysis of target gene expression under specific experimental conditions.

Buffalobur is closely related to tomato, whose qPCR reference genes have been reported in numerous studies^12,34^. *TIP41* has exhibits highly stable expression in different tissues (root, leaves, and inflorescence) at different developmental stages in tomato^12^; however, in buffalobur, it ranks behind other reference genes in different tissues of the fruiting period and leaves of different developmental stages (Table 2). It showed that reference genes used in tomato might not be suitable in genetically related species. Our results here further emphasize the importance of species-specific screening of proper reference genes because genetic difference, even in closely related species, may contribute to distinct and variable expression of these genes under the same experimental conditions.

*P5CS* is stress-responsive as it is involved in the synthesis of key enzymes in the proline synthesis pathway^18^. *GI* participates in developmental processes such as plant flowering, but it is also involved in mediating cold stress and salt stress responses^19^. Under stress conditions such as salinity, drought and ABA, the expression of *P5CS* and *GI* are expected to increase or hold stable rather than decrease to help plants adapt to the negative conditions. In our research, the *P5CS* or *GI* expression levels normalized using the best reference gene combination were significantly different from those normalized with the least stable reference gene (Figs 2 and 3). With the best gene combination for normalization, the expression of *P5CS* and *GI* were only slightly different before and after stresses; with the least stable reference gene, *P5CS* and *GI* expression rose or fell sharply (Figs 2 and 3). Therefore, the reference genes selected in this study are reliable.

In summary, 14 candidate reference genes were first selected under different treatments in buffalobur. For leaves of different developmental stages and leaves of glyphosate-treated plants, the best reference genes are *ACT* and *EF1α*. For different tissues in the fruiting period, the most stable gene pair is *SAND* and *PP2Acs*. *EF1α* and *RPL8* are the most stable reference genes for the abiotic stress group. *EF1α* and *SAND* are suitable reference genes for the hormone stimuli group. For the total samples group, the *EF1α*, *ACT* and *SAND* triplet should be used as reference genes for normalization. This study will facilitate the study of gene expression analysis in buffalobur, which might lay a fundamental path for exploring the molecular mechanism underlying its developmental regulation and for effectively controlling this invasive weed species.

## Materials and Methods

### Plant materials and stress treatments

Buffalobur seeds were collected from the Miyun District (N40.24.082, E116.50.364), Beijing, China, in 2017. Seeds were sown in pots (11 cm diameter) filled with Pindstrup substrate (SIA Pindstrup, Balozi, Latvia) and grown in chambers under a 14 h light/10 h dark photoperiod with 300 μmol·m^−2^·s^−1^ of light intensity at 30 ± 2 °C. When the seedlings were at the 5-leaf stage, they were subjected to three types of stress treatments including abiotic stress (drought, salinity, cold, and heat), hormone stimuli (ABA and GA) and herbicide treatment (glyphosate) (Table 5). For salt or drought treatments, approximately 150 mL of NaCl (300 mM) or 20% PEG-6000 was applied to irrigate the plants; for cold or heat treatments, the plants were transferred to chambers at a temperature of 4 °C or 40 °C under the same photoperiod and light intensity as previously specified; for hormone treatment, the leaves were sprayed with 0.35 mM ABA (Sinopharm Chemical Reagent Co., Ltd., Shanghai, China) or 0.35 mM GA (Sinopharm Chemical Reagent Co., Ltd., Shanghai, China); and for glyphosate treatment, 1680 g ae ha^−1^ of glyphosate (Roundup, isopropylamine salt of glyphosate, 410 g ae L^−1^, Monsanto Company, St. Louis, USA) was sprayed on the leaves. The fifth leaves were collected at 0 (untreated), 24, 48 and 72 h after treatment. Three independent biological replicates per treatment were collected, immediately frozen in liquid nitrogen and stored at −80 °C until RNA extraction.

### Developmental tissue samples

Buffalobur seeds were sown in pots and grown in chambers under the conditions mentioned above. The leaves were collected at different developmental stages; the collected leaves included the cotyledons (cotyledon stage) and the fully expanded 3^rd^ (seedling stage), 5^th^ (vegetative stage) and 7^th^ (fruiting period) leaves (Table 5).

For different tissues in the fruiting period, the roots, stems (6 cm above the root), 7^th^ leaves, petals (without stamens and pistils), and pericarps (with thorns) were collected (Table 5). Three independent biological replicates per treatment were collected, and the storage procedure was same as that for the stress-treatment samples.

### RNA extraction and cDNA synthesis

Total RNA isolation was conducted using the common method described by Chen *et al*.^35^. The concentration and purity of total RNA were quantified using the NanoDrop™ One/OneC ultra trace UV spectrophotometer (ThermoFisher, Waltham, MA, USA). The A260/A280 values of the RNA samples ranged from 1.90 to 2.10. The RNA integrity was assessed using 1% agarose electrophoresis gels stained with Solargel Red nucleic-acid dyes (Solarbio, Beijing, China), and gels of all RNA samples exhibited sharp and intense bands for *28S* and *18S* (see Supplementary Fig. S5). cDNA synthesis was performed with 800 ng of total RNA in a final volume of 20 μL using the same kit described by Chen *et al*., and the synthetic cDNA was stored at −80 °C until use^35^.

### Primer design and qPCR assay

Fourteen candidate reference genes (*GAPDH*, *ACT*, *GR*, *UBQ*, *TIP41*, *RPL8*, *eIF*, *TUB*, *DNAJ*, *CYP*, *EF1α*, *PP2Acs*, *RUBP*, and *SAND*) were selected for the stable expression assay, and the GenBank accession numbers of these genes are MG930815, MG930814, MG930816, MG930817, KT807935, KT807936, KT596731, KT807934, KT596730, MK181638, MK181640, MK181639, MK181641 and MK181642, respectively. The primers for qPCR were designed using the Oligo 7 software; information on the primers is presented in Table 1. Polymerase chain reaction (PCR) was performed to confirm the 14 candidate reference gene sequences, and the primers amplified a single correct target product without visible primer dimers (see Supplementary Fig. S6). The qPCR reactions were performed using the 7500 RealTime PCR System (Applied Biosystems, Waltham, MA, USA) for thermal cycling and SYBR Green detection chemistry (Applied Biosystems, CA, USA). The reaction mixtures and cycling conditions were based on the method described by Chen *et al*.^35^. A single peak was detected on the melting curve for each primer pair after qPCR, which further demonstrated the specificity of these primers (see Supplementary Fig. S7). To confirm the specificity of the primer pairs, the amplification efficiency (E) and correlation coefficient (R^2^) parameters were obtained from the standard curves using the common method^33^. The amplification efficiencies of 14 candidate reference genes ranged from 90.0% to 104.7%, and the correlation coefficients (R^2^) were between 0.990 and 0.998 (Table 1; see Supplementary Fig. S8).

**Table 1:** Information on the selected primer pairs and amplification characteristics of the 14 candidate reference genes and 2 target genes for buffalobur.

Gene symbol	Gene description	Primer sequence F/R	Product length (bp)	Efficiency (%)	Correlation coefficient
*GAPDH*	Glyceraldehyde-3-phosphate dehydrogenase	GCAGTCAACGATCCATTTATCTCCAC TCAACAACGAACTCAGCACCAG	203	101.1	0.995
*ACT*	β-actin	GTGTTCCCTAGCATTGTTGGTCG GCCATATCTTCTCCATATCATCCCAGTTG	172	95.0	0.998
*GR*	Glutaredoxin protein	GCTACTGAGGCTTCCAACAATAACG ACCATAAATTAGCAAGAAAATCACAGAGGC	96	90.0	0.995
*UBQ*	Ubiquitin	GCACTTCTTTTTCCTCTCATTCTCTCG ATGCCTTCCTTGTCTTGAATCTTAGC	168	91.3	0.996
*TIP41*	Tonoplast intrinsic protein/aquaporin	ATCACCCCAGTTCACACCTTAGC GCCCCAACAACAAGCCCAGTTAG	170	91.4	0.994
*RPL8*	Ribosomal protein L8	CAAATCCCACACCCACCACC GCAACACATTACCAACCATAAGACTAGC	260	90.4	0.994
*eIF*	Eukaryotic initiation factor	TGGTCACATCGTCATTAAAAATCGTCCT GGTATCATCAGTTGGGAGCCTCAAG	277	93.1	0.994
*TUB*	β-tubulin	GGTGTTACTTGCTGTTTGAGATTCCCT ATCATCTGTTCATCTACCTCCTTTGTGC	290	104.7	0.990
*DNAJ*	DnaJ-like protein (hsp40)	GTTTCCGCCTCTTGCTCCACA CCGCCGACGAATTTTGCTG	193	96.2	0.991
*CYP*	Cyclophilin	TCAAGAAGGTGGAGGCTGTTGG GACAAGACCCGACCCAAGCA	211	98.6	0.998
*EF1α*	Elongation factor 1-alpha	CTGTGCTGTCCTGATTATTGACTCG GGGTGAAAGCAAGCAACGCA	98	90.6	0.995
*PP2Acs*	A catalytic subunit of protein phosphatase 2A	GCCAGTAAAAAGCCCTGTGACTA CGCAAGCATTCATCATAGAACCCAT	267	97.7	0.997
*RUBP*	Ribulose 1,5 bisphosphate	GCAAAACACTGACATCACCTCCA ATACAAATCCGTGCTCAGTCTCG	206	94.2	0.998
*SAND*	SAND protein family	ACTAGAGAATCGTCAGAGAGGTTTGC CGGAGTAACCCAGCACAGTAGA	267	91.6	0.997
*P5CS*	Delta 1-pyrroline-5-carboxylate synthetase	AGTTCTGTTGAGTGATGTAGAGGGTC CCGATGAAAGAGGGTGCCGAT	271	100.9	0.995
*GI*	GIGANTEA	ACACTACAACCGCCCGATTTT CCATAACACCGCCACACCAACG	233	104.7	0.998

**Table 2:** Gene expression stability ranked by four algorithms for the “Developmental stages” and “Tissues” groups.

Group	Rank	geNorm		NormFinder		BestKeeper			RefFinder	
		Gene	Stability	Gene	Stability	Gene	SD [±Cq]	CV (%Cq)	Gene	Stability
Developmental stages^a^	1	*eIF*	0.343	*ACT*	0.084	*GR*	0.30	1.24	*ACT*	2.11
	2	*SAND*	0.343	*EF1α*	0.124	*ACT*	0.33	1.80	*EF1α*	2.51
	3	*UBQ*	0.411	*GR*	0.185	*PP2Acs*	0.38	1.73	*GR*	2.82
	4	*EF1α*	0.530	*PP2Acs*	0.287	*CYP*	0.44	2.51	*SAND*	3.66
	5	*ACT*	0.576	*CYP*	0.295	*EF1α*	0.48	2.70	*PP2Acs*	4.56
	6	*PP2Acs*	0.610	*SAND*	0.359	*SAND*	0.58	2.45	*CYP*	5.03
	7	*GR*	0.654	*UBQ*	0.425	*UBQ*	0.61	3.10	*eIF*	5.20
	8	*CYP*	0.686	*GAPDH*	0.455	*GAPDH*	0.69	3.74	*UBQ*	5.86
	9	*GAPDH*	0.715	*eIF*	0.522	*eIF*	0.76	3.52	*GAPDH*	7.97
	10	*RPL8*	0.747	*RPL8*	0.564	*RPL8*	0.83	4.03	*RPL8*	10.00
	11	*TUB*	0.794	*TUB*	0.714	*RUBP*	0.88	6.73	*TUB*	11.24
	12	*TIP41*	0.929	*TIP41*	1.040	*TUB*	1.11	5.49	*TIP41*	12.24
	13	*RUBP*	1.039	*RUBP*	1.048	*TIP41*	1.14	5.20	*RUBP*	12.47
	14	*DNAJ*	1.260	*DNAJ*	1.724	*DNAJ*	1.99	9.98	*DNAJ*	14.00
Tissues^b^	1	*eIF*	0.413	*SAND*	0.166	*PP2Acs*	0.40	1.89	*SAND*	1.57
	2	*SAND*	0.413	*PP2Acs*	0.181	*SAND*	0.45	1.94	*PP2Acs*	1.93
	3	*CYP*	0.459	*CYP*	0.289	*CYP*	0.54	3.07	*eIF*	2.51
	4	*EF1α*	0.532	*eIF*	0.364	*GR*	0.56	2.26	*CYP*	3.22
	5	*RPL8*	0.574	*ACT*	0.579	*eIF*	0.67	3.21	*EF1α*	6.45
	6	*GAPDH*	0.601	*GAPDH*	0.619	*UBQ*	0.68	3.88	*GAPDH*	6.51
	7	*PP2Acs*	0.636	*UBQ*	0.640	*ACT*	0.72	4.14	*ACT*	6.85
	8	*UBQ*	0.732	*GR*	0.643	*EF1α*	0.87	5.00	*UBQ*	7.20
	9	*ACT*	0.792	*EF1α*	0.657	*TUB*	1.01	5.17	*GR*	7.52
	10	*GR*	0.843	*RPL8*	0.726	*GAPDH*	1.04	5.74	*RPL8*	8.39
	11	*TUB*	0.943	*TUB*	0.943	*RPL8*	1.06	5.11	*TUB*	10.46
	12	*TIP41*	1.084	*TIP41*	1.206	*DNAJ*	1.52	8.05	*TIP41*	12.24
	13	*DNAJ*	1.330	*DNAJ*	1.483	*TIP41*	1.66	7.70	*DNAJ*	12.74
	14	*RUBP*	1.936	*RUBP*	3.812	*RUBP*	3.85	20.82	*RUBP*	14.00

^a^The fully expanded leaves were harvested at different developmental stages. ^b^Different tissues were harvested in the fruiting period.

**Table 3:** Gene expression stability ranked by four algorithms for the “Abiotic stresses”, “Hormone stimuli” and “Glyphosate” groups.

Group	Rank	geNorm		NormFinder		BestKeeper			RefFinder	
		Gene	Stability	Gene	Stability	Gene	SD [±Cq]	CV (%Cq)	Gene	Stability
Abiotic stresses	1	*RPL8*	0.355	*EF1α*	0.123	*EF1α*	0.42	2.43	*EF1α*	1.00
	2	*EF1α*	0.355	*TUB*	0.175	*TUB*	0.49	2.57	*RPL8*	2.63
	3	*ACT*	0.413	*ACT*	0.201	*PP2Acs*	0.51	2.25	*TUB*	2.83
	4	*TUB*	0.454	*RPL8*	0.207	*RPL8*	0.56	2.82	*ACT*	3.22
	5	*PP2Acs*	0.489	*PP2Acs*	0.245	*SAND*	0.56	2.35	*PP2Acs*	4.40
	6	*SAND*	0.568	*GR*	0.442	*ACT*	0.57	3.06	*SAND*	5.96
	7	*GR*	0.647	*SAND*	0.444	*RUBP*	0.69	5.13	*GR*	6.96
	8	*RUBP*	0.721	*RUBP*	0.585	*GR*	0.78	3.21	*RUBP*	7.97
	9	*CYP*	0.780	*CYP*	0.605	*eIF*	0.85	4.07	*CYP*	8.97
	10	*GAPDH*	0.865	*eIF*	0.780	*CYP*	0.86	4.89	*eIF*	9.97
	11	*eIF*	0.958	*GAPDH*	0.880	*GAPDH*	1.20	6.47	*GAPDH*	10.74
	12	*UBQ*	1.069	*UBQ*	1.056	*DNAJ*	1.24	6.45	*UBQ*	12.24
	13	*TIP41*	1.180	*DNAJ*	1.211	*UBQ*	1.26	6.73	*DNAJ*	13.22
	14	*DNAJ*	1.283	*TIP41*	1.225	*TIP41*	1.47	6.38	*TIP41*	13.49
Hormone stimuli	1	*ACT*	0.250	*SAND*	0.061	*SAND*	0.18	0.78	*SAND*	1.78
	2	*RPL8*	0.250	*EF1α*	0.077	*EF1α*	0.20	1.22	*EF1α*	1.86
	3	*EF1α*	0.264	*ACT*	0.139	*UBQ*	0.28	1.42	*ACT*	2.82
	4	*GAPDH*	0.281	*UBQ*	0.143	*eIF*	0.29	1.41	*RPL8*	3.50
	5	*SAND*	0.300	*RPL8*	0.189	*TUB*	0.31	1.62	*UBQ*	4.12
	6	*UBQ*	0.314	*TUB*	0.216	*RPL8*	0.34	1.78	*TUB*	6.19
	7	*TUB*	0.348	*GAPDH*	0.224	*ACT*	0.35	1.92	*GAPDH*	6.56
	8	*eIF*	0.374	*eIF*	0.254	*GR*	0.36	1.46	*eIF*	6.73
	9	*PP2Acs*	0.393	*PP2Acs*	0.262	*PP2Acs*	0.37	1.66	*PP2Acs*	9.00
	10	*CYP*	0.418	*CYP*	0.274	*CYP*	0.40	2.37	*CYP*	10.00
	11	*GR*	0.443	*GR*	0.341	*GAPDH*	0.40	2.25	*GR*	10.16
	12	*RUBP*	0.484	*RUBP*	0.413	*RUBP*	0.49	3.57	*RUBP*	12.00
	13	*DNAJ*	0.586	*DNAJ*	0.804	*DNAJ*	0.77	4.01	*DNAJ*	13.00
	14	*TIP41*	0.697	*TIP41*	0.906	*TIP41*	0.81	3.56	*TIP41*	14.00
Glyphosate	1	*RPL8*	0.363	*TUB*	0.088	*PP2Acs*	0.26	1.17	*ACT*	2.06
	2	*EF1α*	0.363	*ACT*	0.129	*SAND*	0.30	1.31	*EF1α*	2.51
	3	*ACT*	0.436	*RPL8*	0.134	*ACT*	0.31	1.67	*RPL8*	3.00
	4	*GAPDH*	0.489	*CYP*	0.184	*EF1α*	0.46	2.67	*TUB*	4.12
	5	*CYP*	0.518	*EF1α*	0.194	*CYP*	0.49	2.73	*PP2Acs*	4.30
	6	*TUB*	0.544	*GAPDH*	0.306	*GR*	0.52	2.11	*CYP*	4.47
	7	*PP2Acs*	0.611	*PP2Acs*	0.369	*eIF*	0.56	2.75	*SAND*	5.03
	8	*SAND*	0.648	*SAND*	0.425	*TUB*	0.60	2.99	*GAPDH*	6.78
	9	*GR*	0.687	*GR*	0.591	*RPL8*	0.61	3.05	*GR*	8.13
	10	*eIF*	0.756	*UBQ*	0.668	*UBQ*	0.64	3.41	*eIF*	9.59
	11	*UBQ*	0.792	*eIF*	0.701	*GAPDH*	0.67	3.58	*UBQ*	10.24
	12	*DNAJ*	0.971	*RUBP*	1.299	*DNAJ*	1.39	7.28	*RUBP*	12.49
	13	*RUBP*	1.156	*DNAJ*	1.437	*RUBP*	1.78	11.51	*DNAJ*	12.49
	14	*TIP41*	1.484	*TIP41*	2.338	*TIP41*	2.71	10.45	*TIP41*	14.00

**Table 4:** Gene expression stability ranked by four algorithms for the total samples group.

Rank	geNorm		NormFinder		BestKeeper			RefFinder	
	Gene	Stability	Gene	Stability	Gene	SD [±Cq]	CV (%Cq)	Gene	Stability
1	*ACT*	0.527	*EF1α*	0.275	*SAND*	0.49	2.07	*EF1α*	1.68
2	*PP2Acs*	0.527	*ACT*	0.393	*EF1α*	0.51	2.95	*ACT*	1.86
3	*SAND*	0.712	*SAND*	0.412	*ACT*	0.58	3.16	*SAND*	2.28
4	*EF1α*	0.742	*RPL8*	0.454	*PP2Acs*	0.60	2.67	*PP2Acs*	3.36
5	*RPL8*	0.810	*TUB*	0.462	*GR*	0.60	2.43	*RPL8*	5.14
6	*GR*	0.853	*CYP*	0.466	*TUB*	0.69	3.57	*GR*	5.96
7	*CYP*	0.879	*GR*	0.470	*RPL8*	0.70	3.51	*TUB*	6.62
8	*TUB*	0.907	*PP2Acs*	0.482	*eIF*	0.72	3.43	*CYP*	7.17
9	*GAPDH*	0.937	*eIF*	0.643	*CYP*	0.73	4.17	*eIF*	8.97
10	*eIF*	0.977	*GAPDH*	0.654	*GAPDH*	0.93	5.08	*GAPDH*	9.74
11	*UBQ*	1.051	*UBQ*	0.907	*UBQ*	1.05	5.60	*UBQ*	11.00
12	*DNAJ*	1.179	*TIP41*	1.564	*DNAJ*	1.24	6.46	*DNAJ*	12.00
13	*TIP41*	1.370	*DNAJ*	1.173	*TIP41*	1.75	7.57	*TIP41*	13.00
14	*RUBP*	1.644	*RUBP*	2.162	*RUBP*	1.83	12.51	*RUBP*	14.00

**Table 5:** The summary of samples collected under different conditions in this study.

Stress treatments^a^			Times after treatment							
			0 h^b^ (N = 3)		24 h (N = 3)		48 h (N = 3)			72 h (N = 3)
Abiotic Stresses	Drought		√		√		√			√
	Salt		√		√		√			√
	Cold		√		√		√			√
	Heat		√		√		√			√
Hormone stimuli	ABA		√		√		√			√
	GA		√		√		√			√
Glyphosate			√		√		√			√
**Tissues** ^**c**^										
Roots	Stems		The 7^th^ leaves			Petals			Pericarps	
**Periods** ^**d**^										
Cotyledon stage		Seedling stage		Vegetative stage				Fruiting period		

^a^The 5^th^ leaves were harvested at different time under different kinds of stresses. Three samples were taken at each time point for biological replicates. For the “Drought”, “Salt”, “Cold”, “Heat”, “ABA”, “GA” and “Glyphosate” groups, and each group contains 12 samples (four timepoints, and 3 samples for each timepoint); The “Abiotic Stresses” and “Hormone stimuli” groups contain 48 and 24 samples, respectively. ^b^Leaves without stress treatment were harvested. ^c^Different tissues were harvested in the fruiting period. Three samples were taken from each tissue for biological replicates. ^d^The fully expanded leaves were harvested at different developmental stages. Three samples were taken at each period for biological replicates.

### Data analysis for expression stability

We chose three statistical software programs (geNorm^8^, NormFinder^14^, and BestKeeper^15^) and a web tool (RefFinder^16^) (http://150.216.56.64/referencegene.php?type=reference#) to evaluate the stability of 14 candidate reference genes. The analysis methods of these four programs were the same as those reported in other published articles^35^. geNorm identifies the best reference gene pair by calculating the value M^8^. The smaller the M value, the more stable the gene expression. Furthermore, geNorm also determines the optimal number of reference genes needed by calculating the pairwise variation (V_n_/V_n+1_)^35^. NormFinder evaluates gene expression stability via grouped analysis. The output is not simply the best gene but also the best combination of two genes. The value of expression stabilities derived from NormFinder is smaller, and the single reference gene is more stable. BestKeeper employs pair-wise correlation analysis of all pairs of candidate reference genes to estimate gene expression stability. Pfaffl considers genes with elevated SD or CV values inconsistent^15^. RefFinder generates a comprehensive ranking synthesized using the results of four algorithms (geNorm, NormFinder, BestKeeper and Delta- Ct).

### Validation of reference genes

To validate the reliability of the obtained reference genes, the expression of two target genes—delta 1-pyrroline-5-carboxylate synthetase (*P5CS*) and GIGANTEA (*GI*)—under different experimental conditions was normalized using the combination of the two best reference genes and the most variable gene obtained via RefFinder (see Supplementary Tables S9, S10 and S11). The GenBank accession numbers of *P5CS* and *GI* are MK181643 and MK181644, respectively. Information on the primers for these two target genes is listed in Table 1. The expression levels of these two target genes normalized to the reference genes were analysed using the 2^−ΔΔCt^ method^36^. We set up three biological and technical replicates in qPCR assays.

## Supplementary information

Supplementary Information

## Data Availability

The datasets generated and analyzed during the current study are available from the corresponding author on reasonable request.
