# Supplementary material for: Selection of reference genes for qPCR normalization in buffalobur (Solanum rostratum Dunal)
Source: Sci Rep. 2019 May 6;9:6948. doi: 10.1038/s41598-019-43438-6 (PMC6502881; doi:10.1038/s41598-019-43438-6)
Supplement: Supplementary file 1 — Supplementary Information [file 41598_2019_43438_MOESM1_ESM.pdf]

Title Page

The title:

Selection of reference genes for qPCR normalization in buffalobur (*Solanum rostratum* Dunal)

Author name:

Dandan Zhao[1¶], Xu Wang[1¶], Jingchao Chen[1¶], Zhaofeng Huang[1], Heqiang Huo[2], Cuilan Jiang[1], Hongjuan Huang[1*], Chaoxian Zhang[1] & Shouhui Wei[1*]

Institution and/or department:

[1] Institute of Plant Protection, Chinese Academy of Agricultural Sciences, Beijing, China

[2] Mid-Florida Research and Education Center, University of Florida, Institute of Food and Agricultural Sciences, Apopka, Florida, United States of America

*** Corresponding author:**

Correspondence: Shouhui Wei and Hongjuan Huang.

Institute of Plant Protection, Chinese Academy of Agricultural Sciences, Beijing, China

Tel& Fax: 86-010-62815937

E-mail: shweiippcaas@126.com (SW), hjhuang@ippcaas.cn (HH)

[¶]These authors contributed equally to this work.

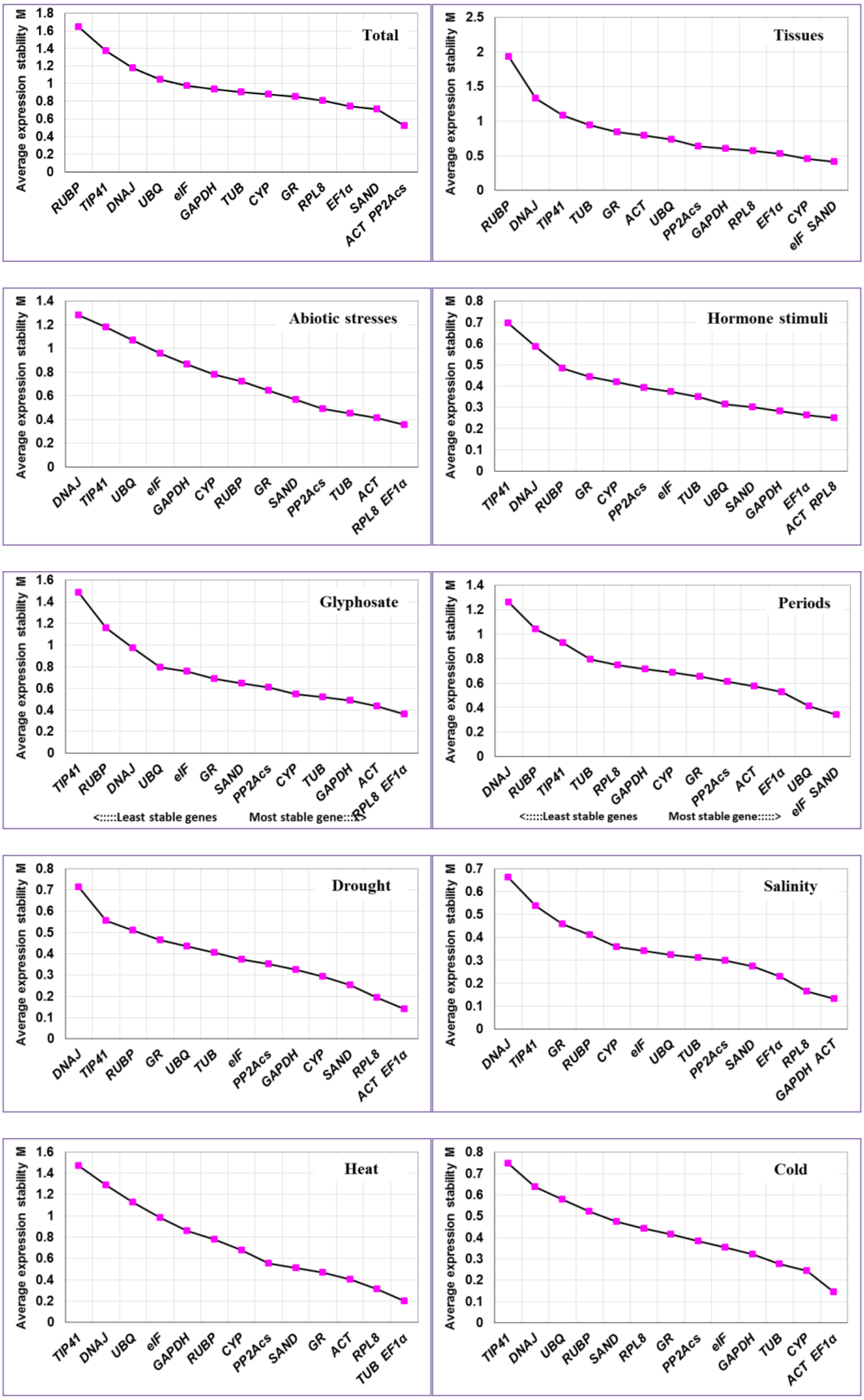
Total
Average expression stability M
RUBP TIP41 DNAJ UBQ eIF GAPDH TUB CYP GR RPL8 EF1α SAND ACT PP2Acs
Tissues
Average expression stability M
RUBP DNAJ TIP41 TUB GR ACT UBQ PP2Acs GAPDH RPL8 EF1α CYP eIF SAND
Abiotic stresses
Average expression stability M
DNAJ TIP41 UBQ eIF GAPDH CYP RUBP GR SAND PP2Acs TUB ACT RPL8 EF1α
Hormone stimuli
Average expression stability M
TIP41 DNAJ RUBP GR CYP PP2Acs eIF TUB UBQ SAND GAPDH EF1α ACT RPL8
Glyphosate
Average expression stability M
TIP41 RUBP DNAJ UBQ eIF GR SAND PP2Acs CYP TUB GAPDH ACT RPL8 EF1α
<:::::Least stable genes
Most stable gene:::::>
Periods
Average expression stability M
DNAJ RUBP TIP41 TUB RPL8 GAPDH CYP GR PP2Acs ACT EF1α UBQ eIF SAND
<:::::Least stable genes
Most stable gene:::::>
Drought
Average expression stability M
DNAJ TIP41 RUBP GR UBQ TUB eIF PP2Acs GAPDH CYP SAND RPL8 ACT EF1α
Salinity
Average expression stability M
DNAJ TIP41 GR RUBP CYP eIF UBQ TUB PP2Acs SAND EF1α RPL8 GAPDH ACT
Heat
Average expression stability M
TIP41 DNAJ UBQ eIF GAPDH RUBP CYP PP2Acs SAND GR ACT RPL8 TUB EF1α
Cold
Average expression stability M
TIP41 DNAJ UBQ RUBP SAND RPL8 GR PP2Acs eIF GAPDH TUB CYP ACT EF1α

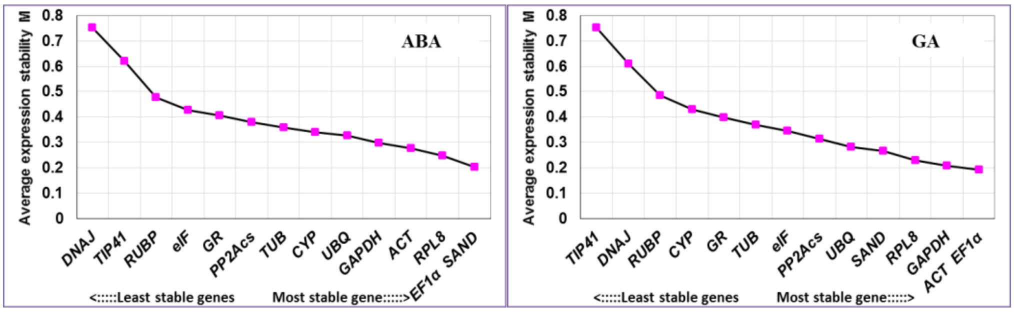

Supplementary Figure S1. Average expression stability values (M) calculated using geNorm.

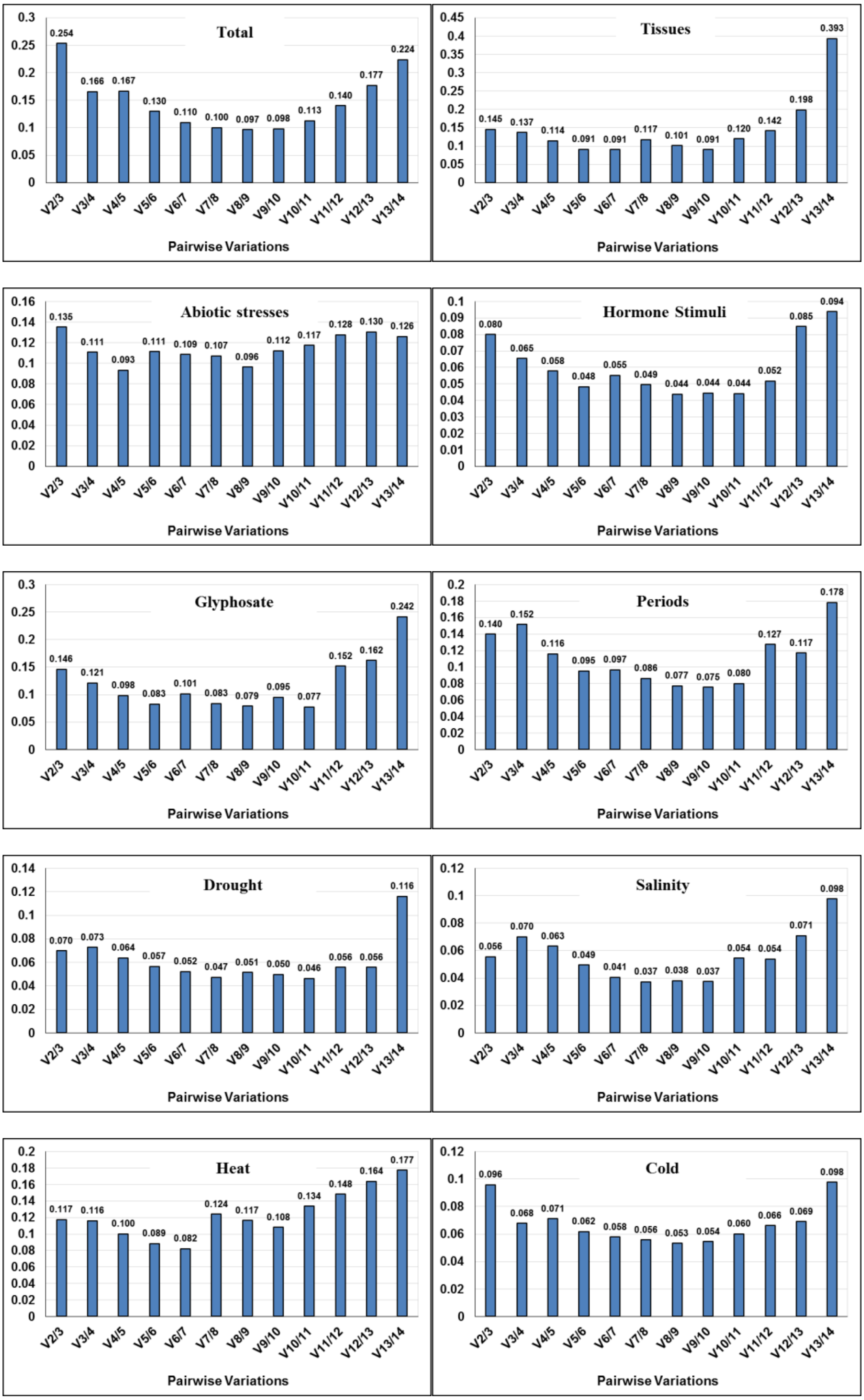
Total
0.254
0.166
0.167
0.130
0.110
0.100
0.097
0.098
0.113
0.140
0.177
0.224
Tissues
0.145
0.137
0.114
0.091
0.091
0.117
0.101
0.091
0.120
0.142
0.198
0.393
Abiotic stresses
0.135
0.111
0.093
0.111
0.109
0.107
0.096
0.112
0.117
0.128
0.130
0.126
Hormone Stimuli
0.080
0.065
0.058
0.048
0.055
0.049
0.044
0.044
0.044
0.052
0.085
0.094
Glyphosate
0.146
0.121
0.098
0.083
0.101
0.083
0.079
0.095
0.077
0.152
0.162
0.242
Periods
0.140
0.152
0.116
0.095
0.097
0.086
0.077
0.075
0.080
0.127
0.117
0.178
Drought
0.070
0.073
0.064
0.057
0.052
0.047
0.051
0.050
0.046
0.056
0.056
0.116
Salinity
0.056
0.070
0.063
0.049
0.041
0.037
0.038
0.037
0.054
0.054
0.071
0.098
Heat
0.117
0.116
0.100
0.089
0.082
0.124
0.117
0.108
0.134
0.148
0.164
0.177
Cold
0.096
0.068
0.071
0.062
0.058
0.056
0.053
0.054
0.060
0.066
0.069
0.098
V2/3
V3/4
V4/5
V5/6
V6/7
V7/8
V8/9
V9/10
V10/11
V11/12
V12/13
V13/14
Pairwise Variations

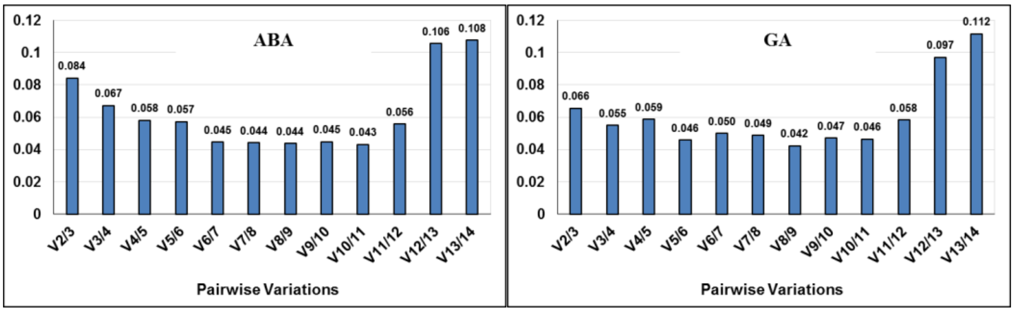

Supplementary Figure S2. Pairwise variation (V) to define the optimal number of reference genes required for reliable normalization in each dataset obtained using geNorm.

Supplementary Table S3. The best combination obtained via NormFinder for different conditions. [a]Abiotic stresses, grouped by stress type (drought, salinity, cold and heat). [b]Hormone stimuli, grouped by ABA and GA. [c]Glyphosate, grouped by time (0, 24, 48 and 72 h). [d]Tissues, grouped by root, leaf, stem, petal and pericarps. [e]Developmental stages, grouped by cotyledon stage, seedling stage, vegetative stage and fruiting period. [f]Drought, grouped by time (0, 24, 48 and 72 h). [g]Salinity, grouped by time (0, 24, 48 and 72 h). [h]Cold, grouped by time (0, 24, 48 and 72 h). [i]Heat, grouped by time (0, 24, 48 and 72 h). [j]ABA, grouped by time (0, 24, 48 and 72 h). [k]GA, grouped by time (0, 24, 48 and 72 h). [l]Total, grouped by subgroup (abiotic stresses, hormone stimuli, glyphosate, tissues and developmental stages).

**Group**	**Best combination of two genes**	**Stability value for best combination of two genes**
Abiotic stresses[a]	*TUB* and *EF1α*	0.036
Hormone stimuli[b]	*EF1α* and *SAND*	0.015
Glyphosate[c]	*eIF* and *CYP*	0.079
Tissues[d]	*GR* and *PP2Acs*	0.084
Periods[e]	*GR* and *eIF*	0.082
Drought[f]	*GAPDH* and *EF1α*	0.067
Salinity[g]	*ACT* and *CYP*	0.066
Cold[h]	*ACT* and *CYP*	0.053
Heat[i]	*GAPDH* and *eIF*	0.063
ABA[j]	*EF1α* and *SAND*	0.062
GA[k]	*ACT* and *EF1α*	0.044
Total[l]	*GAPDH* and *SAND*	0.046

Supplementary Table S4. The coefficient of variation (CV (%)) of *P5CS* and *GI* relative expression normalized using different reference genes under different stress conditions. The good reference genes are the combination of the two best reference genes obtained via RefFinder. The poor reference gene is the least stable gene obtained via RefFinder.

		P5CS					*GI*				
		0h	24h	48h	72h	0-72 h	0h	24h	48h	72h	0-72 h
Glyphosate	Good normalisation	3.24	7.26	7.96	12.54	32.73	8.86	11.02	4.22	10.58	63.46
	Poor normalisation	28.95	20.34	1.38	14.16	111.62	20.23	21.57	27.02	19.01	137.56
Drought	Good normalisation	3.24	2.49	5.90	17.57	13.46	8.86	9.13	16.35	10.95	22.79
	Poor normalisation	12.59	31.05	25.86	11.55	144.00	7.96	23.35	24.82	18.30	153.55
Salinity	Good normalisation	2.73	8.97	8.66	9.22	19.33	9.10	23.41	16.18	8.66	38.41
	Poor normalisation	12.59	10.79	9.90	28.19	76.43	7.96	18.84	16.49	39.79	93.29
Heat	Good normalisation	3.74	2.09	11.06	13.26	21.39	11.49	7.13	14.29	13.70	56.77
	Poor normalisation	28.95	16.51	4.19	16.46	66.38	20.23	16.10	52.74	22.75	77.89
Cold	Good normalisation	3.24	7.23	11.70	5.92	24.45	8.86	12.39	21.36	20.41	39.38
	Poor normalisation	28.94	18.37	49.40	4.12	82.05	20.23	22.08	38.67	14.77	66.50
ABA	Good normalisation	16.81	6.89	11.46	10.36	16.69	9.76	4.36	12.33	18.46	20.31
	Poor normalisation	12.59	10.82	20.83	24.38	106.16	7.96	10.78	34.10	28.10	124.72
GA	Good normalisation	3.24	26.79	13.36	17.62	21.95	8.86	15.72	13.00	4.05	30.55
	Poor normalisation	28.94	34.45	11.53	25.24	59.19	20.23	15.60	15.60	5.66	42.22

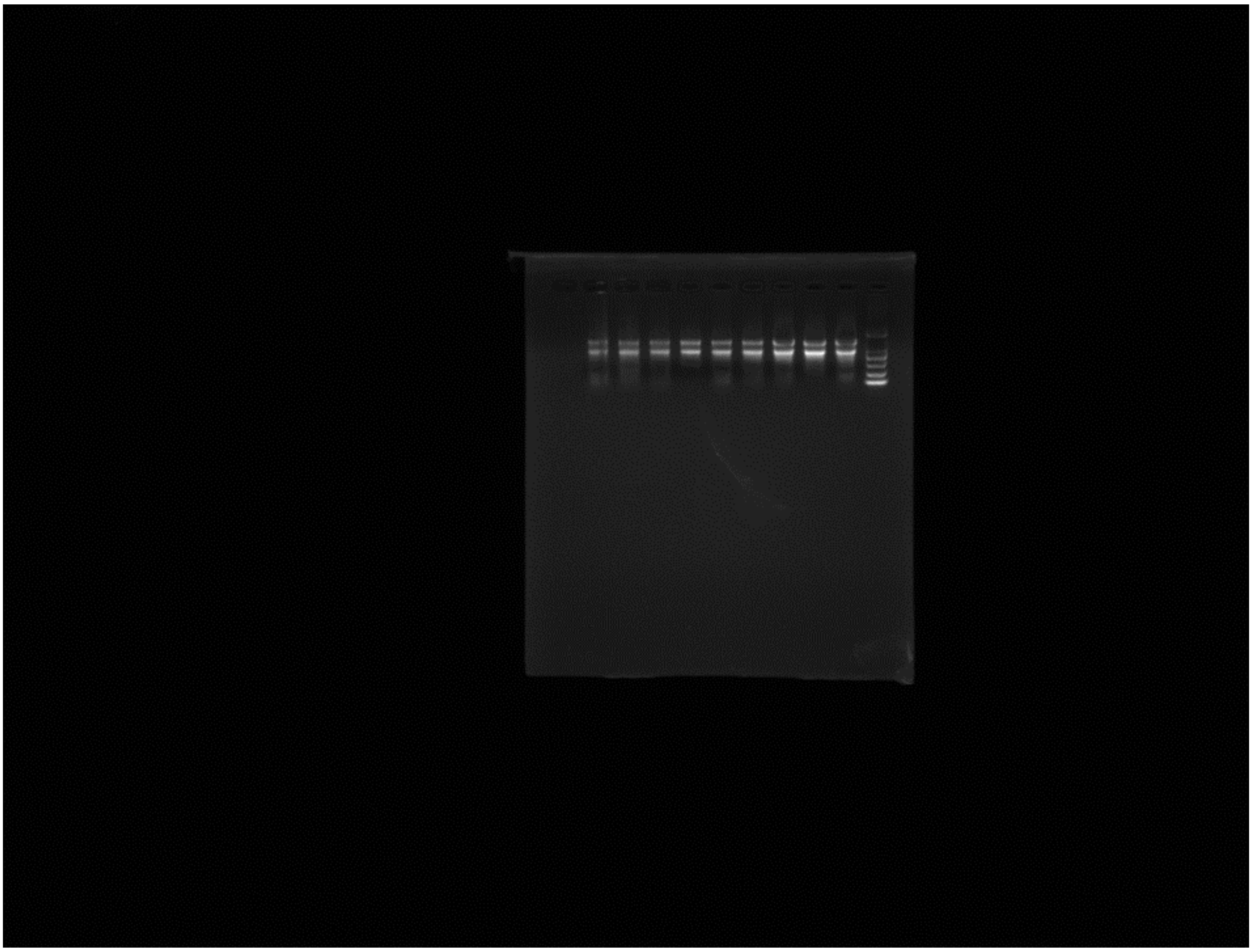

Supplementary Figure S5. Total RNA extracts detected via 1% gel electrophoresis for parts of the samples. M: DL 2000 marker.

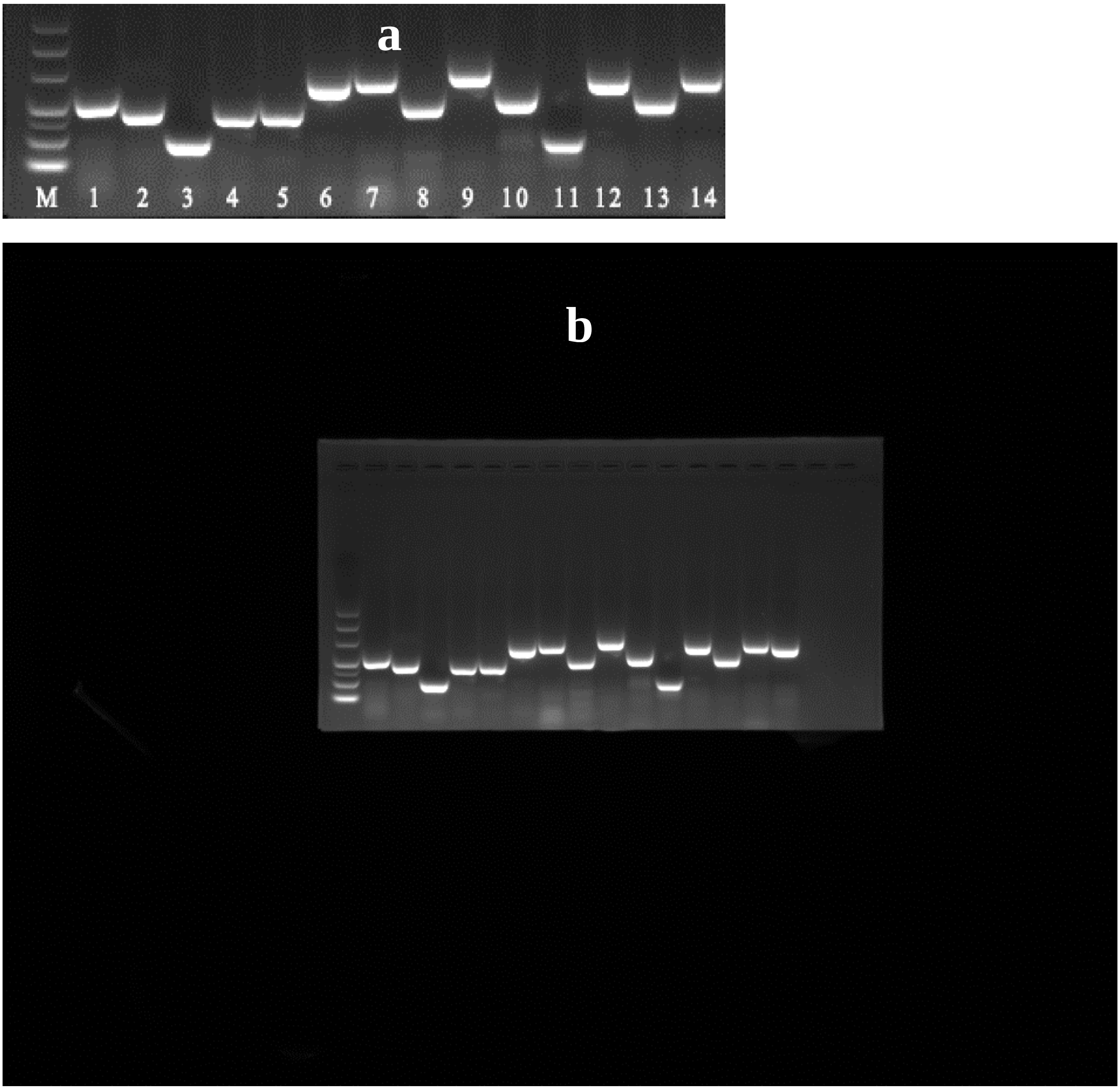

Supplementary Figure S6 (a) Gel electrophoresis of amplification products for 14 candidate reference genes. M: DL500 marker; from 1 to 14: *GAPDH*, *ACT*, *GR*, *UBQ*, *TIP41*, *RPL8*, *eIF*, *TUB*, *DNAJ*, *CYP*, *EF1α*, *PP2Acs*, *RUBP*, and *SAND*. (b) The full-size gels of part a.

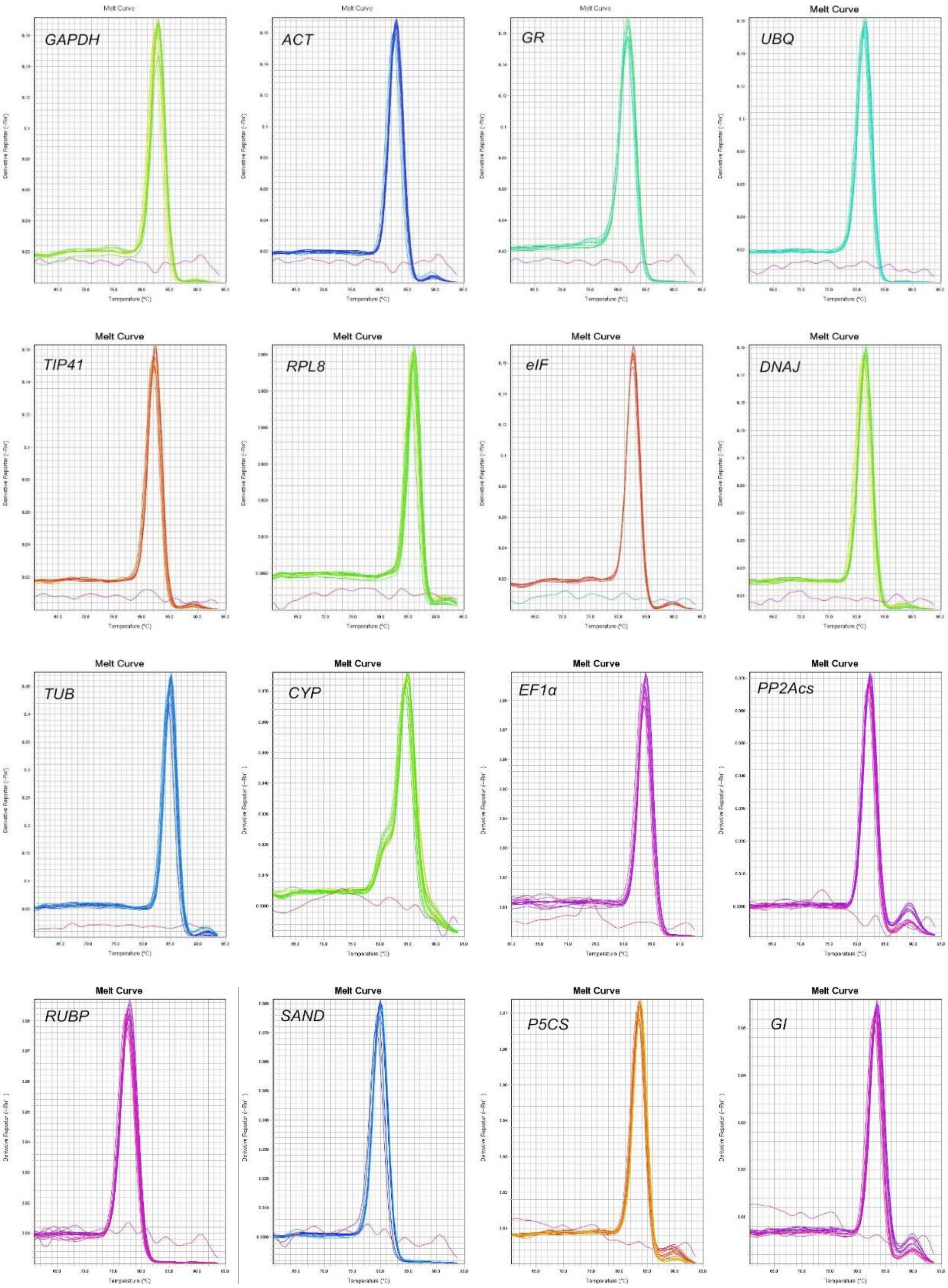

Supplementary Figure S7. Melt curves of 14 candidate reference genes and 2 target genes, obtained through qPCR.

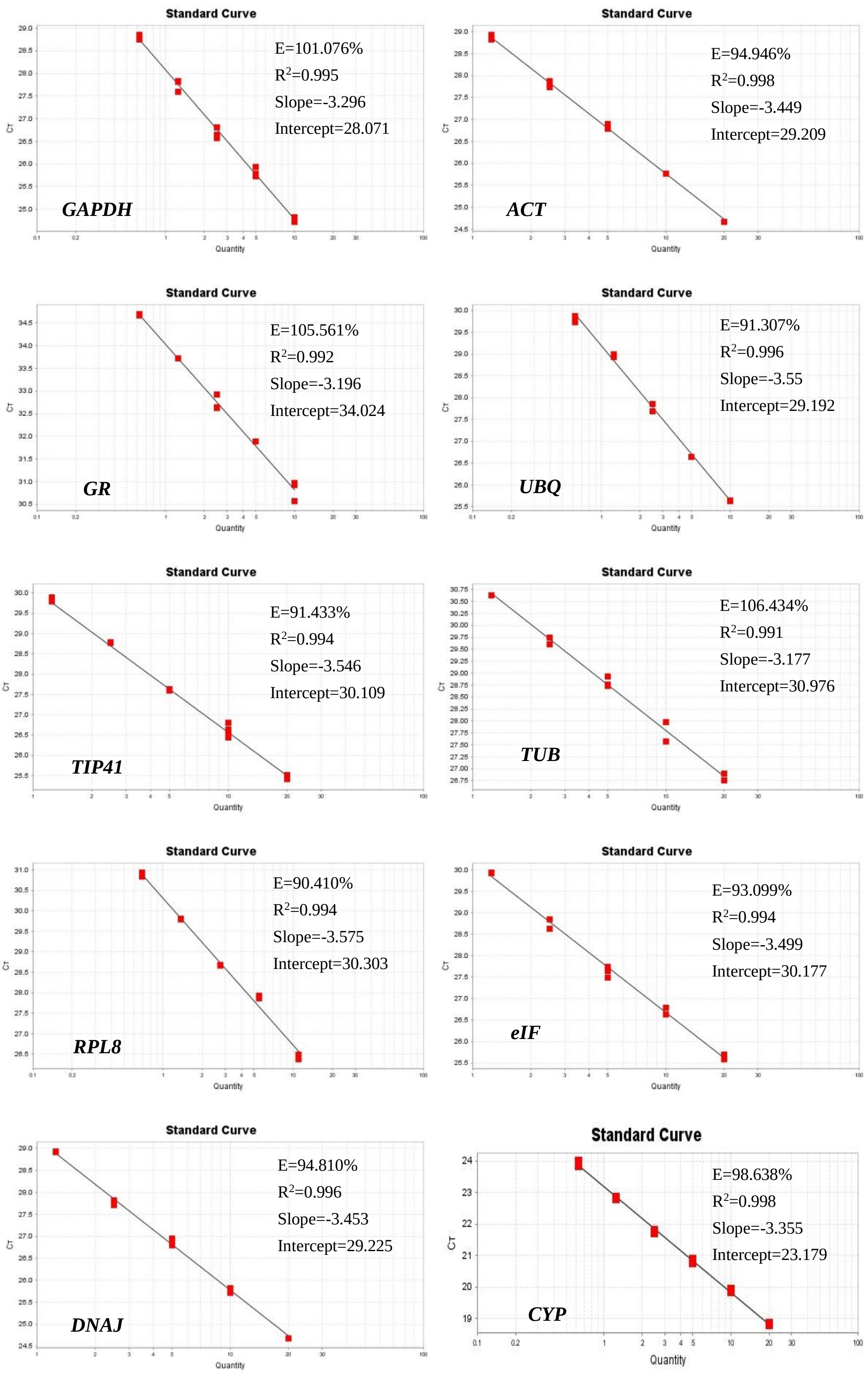
Standard Curve
E=101.076%
R^2=0.995
Slope=-3.296
Intercept=28.071
GAPDH
Quantity
Standard Curve
E=94.946%
R^2=0.998
Slope=-3.449
Intercept=29.209
ACT
Quantity
Standard Curve
E=105.561%
R^2=0.992
Slope=-3.196
Intercept=34.024
GR
Quantity
Standard Curve
E=91.307%
R^2=0.996
Slope=-3.55
Intercept=29.192
UBQ
Quantity
Standard Curve
E=91.433%
R^2=0.994
Slope=-3.546
Intercept=30.109
TIP41
Quantity
Standard Curve
E=106.434%
R^2=0.991
Slope=-3.177
Intercept=30.976
TUB
Quantity
Standard Curve
E=90.410%
R^2=0.994
Slope=-3.575
Intercept=30.303
RPL8
Quantity
Standard Curve
E=93.099%
R^2=0.994
Slope=-3.499
Intercept=30.177
eIF
Quantity
Standard Curve
E=94.810%
R^2=0.996
Slope=-3.453
Intercept=29.225
DNAJ
Quantity
Standard Curve
E=98.638%
R^2=0.998
Slope=-3.355
Intercept=23.179
CYP
Quantity

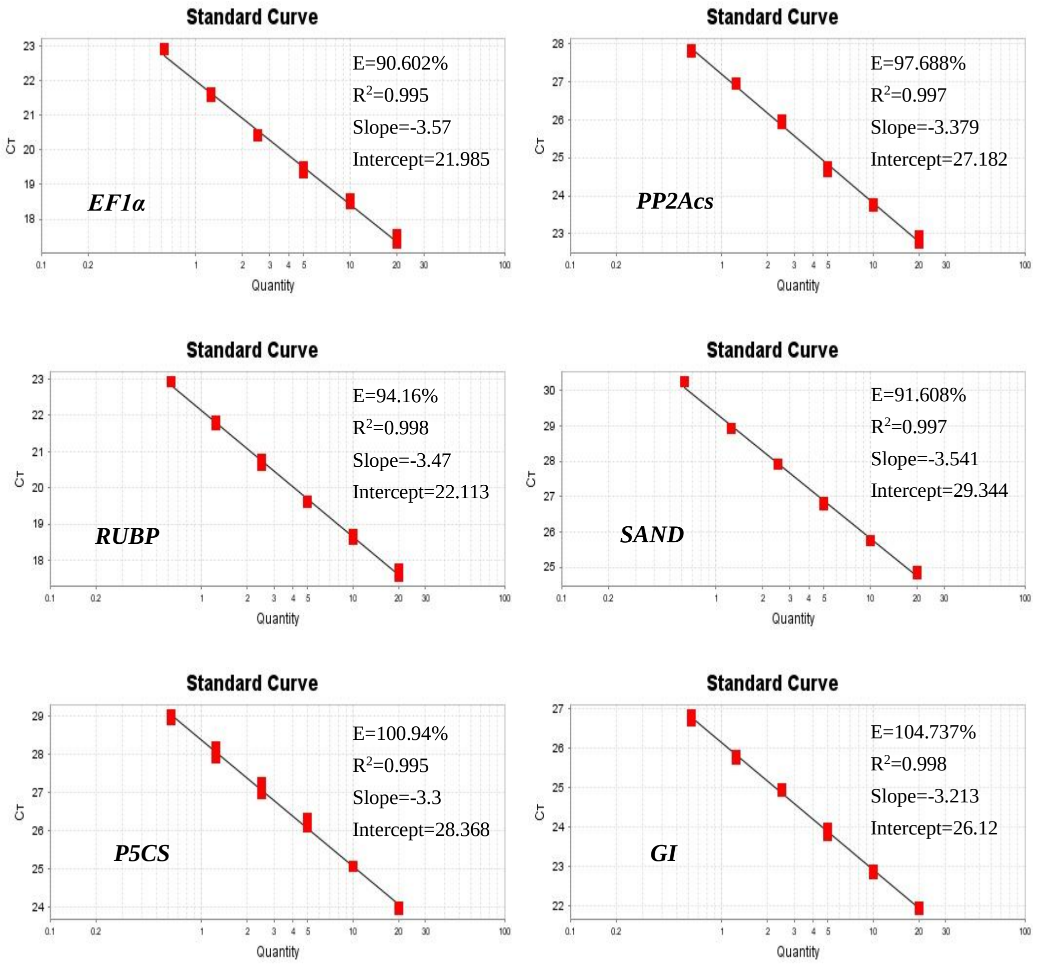

Supplementary Figure S8. Standard curves of 14 candidate reference genes and 2 target genes for qPCR.

Supplementary Table S9. Gene expression stability ranked by four algorithms for drought and salinity stresses.

Group	Rank	geNorm		NormFinder		BestKeeper			RefFinder	
		Gene	Stability	Gene	Stability	Gene	SD[±Cq]	CV(%Cq)	Gene	Stability
Drough	1	*ACT*	0.139	*EF1α*	0.087	*CYP*	0.32	1.85	*EF1α*	1.32
	2	*EF1α*	0.139	*SAND*	0.092	*eIF*	0.33	1.55	*ACT*	2.21
	3	*RPL8*	0.193	*ACT*	0.129	*EF1α*	0.33	1.92	*CYP*	3.64
	4	*SAND*	0.252	*RPL8*	0.179	*ACT*	0.34	1.82	*SAND*	3.72
	5	*CYP*	0.294	*eIF*	0.179	*RPL8*	0.36	1.82	*RPL8*	4.16
	6	*GAPDH*	0.325	*GAPDH*	0.207	*GAPDH*	0.36	1.99	*eIF*	4.76
	7	*PP2Acs*	0.351	*CYP*	0.207	*UBQ*	0.36	1.82	*GAPDH*	6
	8	*eIF*	0.374	*PP2Acs*	0.209	*SAND*	0.37	1.55	*PP2Acs*	7.71
	9	*TUB*	0.404	*UBQ*	0.305	*PP2Acs*	0.4	1.78	*UBQ*	8.91
	10	*UBQ*	0.436	*TUB*	0.311	*RUBP*	0.4	3.03	*TUB*	9.72
	11	*GR*	0.463	*GR*	0.364	*TUB*	0.49	2.53	*GR*	11.24
	12	*RUBP*	0.509	*RUBP*	0.462	*GR*	0.53	2.14	*RUBP*	11.47
	13	*TIP41*	0.554	*TIP41*	0.560	*TIP41*	0.77	3.53	*TIP41*	13
	14	*DNAJ*	0.712	*DNAJ*	1.123	*DNAJ*	1.06	5.49	*DNAJ*	14
Salinity	1	*GAPDH*	0.131	*ACT*	0.073	*eIF*	0.41	1.91	*ACT*	1.41
	2	*ACT*	0.131	*EF1α*	0.099	*CYP*	0.43	2.47	*GAPDH*	2.66
	3	*RPL8*	0.163	*SAND*	0.128	*UBQ*	0.46	2.28	*EF1α*	3.72
	4	*EF1α*	0.228	*UBQ*	0.129	*ACT*	0.5	2.73	*UBQ*	4.43
	5	*SAND*	0.274	*GAPDH*	0.141	*GAPDH*	0.52	2.91	*SAND*	5.1
	6	*PP2Acs*	0.298	*PP2Acs*	0.156	*PP2Acs*	0.53	2.33	*eIF*	5.48
	7	*TUB*	0.31	*TUB*	0.171	*TUB*	0.53	2.72	*PP2Acs*	6
	8	*UBQ*	0.323	*CYP*	0.207	*EF1α*	0.55	3.21	*CYP*	6.16
	9	*eIF*	0.34	*RPL8*	0.207	*SAND*	0.58	2.45	*RPL8*	6.75
	10	*CYP*	0.359	*eIF*	0.241	*RUBP*	0.61	4.59	*TUB*	7.24
	11	*RUBP*	0.41	*GR*	0.406	*RPL8*	0.61	3.12	*RUBP*	11.22
	12	*GR*	0.458	*RUBP*	0.464	*GR*	0.68	2.78	*GR*	11.49
	13	*TIP41*	0.538	*TIP41*	0.672	*TIP41*	0.92	4.18	*TIP41*	13
	14	*DNAJ*	0.662	*DNAJ*	0.944	*DNAJ*	1.31	6.35	*DNAJ*	14

Supplementary Table S10. Gene expression stability ranked by four algorithms for heat and cold stresses.

Group	Rank	geNorm		NormFinder		BestKeeper			RefFinder	
		Gene	Stability	Gene	Stability	Gene	SD[±Cq]	CV(%Cq)	Gene	Stability
Heat	1	*TUB*	0.201	*TUB*	0.070	*TUB*	0.3	1.57	*TUB*	1.19
	2	*EF1α*	0.201	*EF1α*	0.070	*EF1α*	0.33	1.93	*EF1α*	1.41
	3	*RPL8*	0.311	*RPL8*	0.117	*SAND*	0.47	2.04	*RPL8*	3.57
	4	*ACT*	0.404	*SAND*	0.220	*GR*	0.51	2.06	*GR*	4.47
	5	*GR*	0.467	*GR*	0.201	*PP2Acs*	0.55	2.41	*SAND*	4.82
	6	*SAND*	0.51	*ACT*	0.237	*RPL8*	0.56	2.82	*ACT*	5.09
	7	*PP2Acs*	0.551	*PP2Acs*	0.330	*ACT*	0.58	3.07	*PP2Acs*	6.44
	8	*CYP*	0.676	*CYP*	0.710	*RUBP*	0.69	4.84	*CYP*	8.66
	9	*RUBP*	0.776	*RUBP*	0.751	*eIF*	0.87	4.38	*RUBP*	8.74
	10	*GAPDH*	0.857	*eIF*	0.872	*UBQ*	1.1	6.24	*eIF*	10.22
	11	*eIF*	0.981	*GAPDH*	0.877	*CYP*	1.23	6.58	*GAPDH*	10.72
	12	*UBQ*	1.125	*UBQ*	1.215	*GAPDH*	1.25	6.23	*UBQ*	11.47
	13	*DNAJ*	1.289	*DNAJ*	1.577	*DNAJ*	1.55	8.47	*DNAJ*	13
	14	*TIP41*	1.467	*TIP41*	1.709	*TIP41*	2.08	8.49	*TIP41*	14
Cold	1	*ACT*	0.144	*EF1α*	0.079	*ACT*	0.25	1.4	*EF1α*	1.41
	2	*EF1α*	0.144	*ACT*	0.082	*EF1α*	0.28	1.63	*ACT*	1.57
	3	*CYP*	0.244	*CYP*	0.083	*PP2Acs*	0.28	1.25	*CYP*	2.59
	4	*TUB*	0.276	*TUB*	0.093	*TUB*	0.29	1.53	*TUB*	4
	5	*GAPDH*	0.321	*GAPDH*	0.221	*CYP*	0.31	1.86	*GAPDH*	5.23
	6	*eIF*	0.354	*PP2Acs*	0.240	*GAPDH*	0.25	1.4	*PP2Acs*	5.45
	7	*PP2Acs*	0.384	*eIF*	0.242	*eIF*	0.45	2.15	*eIF*	6.48
	8	*GR*	0.415	*RPL8*	0.301	*SAND*	0.49	2.02	*RPL8*	8.49
	9	*RPL8*	0.443	*GR*	0.307	*RPL8*	0.49	2.51	*GR*	8.97
	10	*SAND*	0.475	*SAND*	0.381	*GR*	0.5	2.13	*SAND*	9.46
	11	*RUBP*	0.522	*RUBP*	0.487	*RUBP*	0.63	4.83	*RUBP*	11
	12	*UBQ*	0.577	*UBQ*	0.548	*UBQ*	0.71	3.88	*UBQ*	12
	13	*DNAJ*	0.637	*DNAJ*	0.647	*DNAJ*	0.75	3.7	*DNAJ*	13
	14	*TIP41*	0.747	*TIP41*	0.943	*TIP41*	1.01	4.66	*TIP41*	14

Supplementary Table S11. Gene expression stability ranked by four algorithms for groups of hormone stimuli.

Group	Rank	geNorm		NormFinder		BestKeeper			RefFinder	
		**Gene**	**Stability**	**Gene**	**Stability**	**Gene**	**SD[±Cq]**	**CV(%Cq)**	**Gene**	**Stability**
ABA	1	*EF1α*	0.202	*EF1α*	0.070	*SAND*	0.13	0.55	*SAND*	1
	2	*SAND*	0.202	*SAND*	0.070	*EF1α*	0.13	0.77	*EF1α*	1.68
	3	*RPL8*	0.248	*RPL8*	0.148	*UBQ*	0.22	1.1	*RPL8*	3.22
	4	*ACT*	0.276	*UBQ*	0.157	*RPL8*	0.23	1.19	*UBQ*	4.36
	5	*GAPDH*	0.297	*ACT*	0.162	*TUB*	0.27	1.4	*ACT*	4.68
	6	*UBQ*	0.327	*CYP*	0.169	*ACT*	0.27	1.47	*CYP*	6.48
	7	*CYP*	0.341	*TUB*	0.204	*CYP*	0.27	1.6	*TUB*	6.88
	8	*TUB*	0.359	*GAPDH*	0.204	*PP2Acs*	0.3	1.35	*GAPDH*	7.27
	9	*PP2Acs*	0.381	*PP2Acs*	0.228	*eIF*	0.33	1.56	*PP2Acs*	8.74
	10	*GR*	0.405	*eIF*	0.300	*GAPDH*	0.35	1.91	*eIF*	9.97
	11	*eIF*	0.428	*GR*	0.348	*GR*	0.4	1.63	*GR*	10.74
	12	*RUBP*	0.478	*RUBP*	0.456	*RUBP*	0.59	4.26	*RUBP*	12
	13	*TIP41*	0.621	*TIP41*	1.000	*TIP41*	1.05	4.67	*TIP41*	13
	14	*DNAJ*	0.753	*DNAJ*	1.040	*DNAJ*	1.08	5.56	*DNAJ*	14
GA	1	*ACT*	0.192	*ACT*	0.066	*SAND*	0.21	0.88	*EF1α*	1.32
	2	*EF1α*	0.192	*EF1α*	0.066	*ACT*	0.22	1.21	*ACT*	1.68
	3	*GAPDH*	0.209	*SAND*	0.098	*EF1α*	0.24	1.43	*SAND*	2.59
	4	*RPL8*	0.229	*GAPDH*	0.131	*GAPDH*	0.25	1.41	*GAPDH*	3.72
	5	*SAND*	0.265	*UBQ*	0.149	*UBQ*	0.31	1.59	*UBQ*	5.23
	6	*UBQ*	0.283	*RPL8*	0.149	*PP2Acs*	0.32	1.45	*RPL8*	5.83
	7	*PP2Acs*	0.314	*TUB*	0.209	*GR*	0.33	1.33	*PP2Acs*	7.2
	8	*eIF*	0.346	*PP2Acs*	0.243	*RPL8*	0.33	1.73	*TUB*	7.94
	9	*TUB*	0.368	*eIF*	0.274	*TUB*	0.35	1.89	*eIF*	8.97
	10	*GR*	0.398	*GR*	0.298	*eIF*	0.38	1.81	*GR*	9.15
	11	*CYP*	0.43	*CYP*	0.318	*CYP*	0.46	2.74	*CYP*	11
	12	*RUBP*	0.485	*RUBP*	0.470	*RUBP*	0.5	3.67	*RUBP*	12
	13	*DNAJ*	0.61	*DNAJ*	0.935	*TIP41*	1	4.42	*DNAJ*	13.24
	14	*TIP41*	0.752	*TIP41*	1.080	*DNAJ*	1.01	5.2	*TIP41*	13.74
